# Replication study of polymorphisms associated with response to methotrexate in patients with rheumatoid arthritis

**DOI:** 10.1038/s41598-018-25634-y

**Published:** 2018-05-09

**Authors:** Rosario López-Rodríguez, Aida Ferreiro-Iglesias, Aurea Lima, Miguel Bernardes, Andrzej Pawlik, Agnieszka Paradowska-Gorycka, Jerzy Świerkot, Ryszard Slezak, Vita Dolžan, Isidoro González-Álvaro, Javier Narváez, Rafael Cáliz, Eva Pérez-Pampín, Antonio Mera-Varela, Laura Vidal-Bralo, José Gorgonio Acuña Ochoa, Carmen Conde, Juan J. Gómez-Reino, Antonio González

**Affiliations:** 1 Experimental and Observational Rheumatology and Rheumatology Unit, Instituto de Investigación Sanitaria - Hospital Clínico Universitario de Santiago, Travesia Choupana sn, 15706 Santiago de Compostela, Spain; 2 CESPU, Institute of Research & Advanced Training in Health Sciences & Technologies, Department of Pharmaceutical Sciences, Rua Central de Gandra, 1317, 4585-116 Gandra, PRD Portugal; 3 Faculty of Medicine of University of Porto, Alameda Prof. Hernâni Monteiro, 4200-319 Porto, Portugal; 4 Rheumatology Department of São João Hospital Center, Porto, Portugal; 5 Department of Physiology, Pomeranian Medical University Szczecin, Rybacka 1, 70-204 Szczecin, Poland; 6 Department of Biochemistry and Molecular Biology, National Institute of Geriatrics, Rheumatology and Rehabilitation, Spartańska 1, 02-637 Warsaw, Poland; 7 Department of Rheumatology, Wroclaw Medical University, Ludwika Pasteura 1, 50-367 Wroclaw, Poland; 8 Department of Genetics, Wroclaw Medical University, Ludwika Pasteura 1, 50-367 Wroclaw, Poland; 9 Pharmacogenetics Laboratory, Institute of Biochemistry, Faculty of Medicine, University of Ljubljana, Vrazov Trg 2, 1000 Ljubljana, Slovenia; 10 Rheumatology Department, Instituto de Investigacion del Hospital de La Princesa (IIS-IP), Diego de León 62, 28006 Madrid, Spain; 11 Department of Rheumatology, Hospital Universitario de Bellvitge-IDIBELL, Carrer de la Feixa Llarga, s/n, 08907 Barcelona, Spain; 12 Rheumatology Unit, Hospital Universitario Virgen de las Nieves, Av. de las Fuerzas Armadas, 2, 18014 Granada, Spain

## Abstract

About 70 genetic studies have already addressed the need of biomarkers to predict the response of patients with rheumatoid arthritis (RA) to methotrexate (MTX) treatment. However, no genetic biomarker has yet been sufficiently validated. Here, we aimed to replicate a selection of 25 SNPs in the largest collection of patients up to date, which consisted of 915 patients treated with MTX. The change in disease activity (measured as ΔDAS28) from baseline was considered the primary outcome. In addition, response according to widely used criteria (EULAR) was taken as secondary outcome. We considered consistency between outcomes, *P* values accounting for the number of SNPs, and independence from potential confounders for interpretation of the results. Only the rs1801394 SNP in *MTRR* fulfilled the high association standards. Its minor allele was associated with less improvement than the major allele according to ΔDAS28 (p = 0.0016), and EULAR response (p = 0.004), with independence of sex, age, baseline DAS28, smoking, seropositivity, concomitant corticosteroid use or previous treatments. In addition, previous evidence suggests the association of this SNP with response to MTX in another autoimmune disease, juvenile idiopathic arthritis, and with high intracellular folate levels, which could contribute to poor response.

## Introduction

Methotrexate (MTX) is the most widely prescribed disease-modifying anti-rheumatic drug (DMARD), and MTX monotherapy is the first treatment recommended for most new patients with rheumatoid arthritis (RA)^1,2^. However, a significant decrease in disease activity is not observed at six months of follow-up in a sizable fraction of patients. This variable response calls for biomarkers to predict patients with low chances of benefiting from MTX monotherapy. The biomarkers have been searched specially in the area of pharmacogenetics^3–5^. About 70 studies have explored polymorphisms in candidate genes including the primary target of MTX, the enzyme dihydrofolate reductase (DHFR), which converts dihydrofolate to tetrahydrofolate. Tetrahydrofolate is essential for purine synthesis, and in the biologically active form, 5-methyl-tetrahydrofolate, is an important cofactor in one-carbon metabolism. Candidate genes are involved in the transport of MTX, generation of its active metabolites, MTX polyglutamates, or in folate one-carbon metabolism, purine synthesis, or in the biosynthetic pathway for adenosine whose accumulation could be the main anti-inflammatory effect of MTX treatment^3,4^. However, no reproducible association has emerged yet from these candidate gene studies. This panorama has been completed recently, thanks to the first GWAS, which was performed in RA patients from the North of India^6^. The authors highlighted 7 SNPs with suggestive evidence of association, none of them located in candidate loci, and several SNPs in candidate genes, which were only apparent after specifically looking for them.

Here, we aimed to replicate 25 of the most promising genetic biomarkers previously associated with MTX response. Fourteen were selected from candidate gene studies^3,4,7–21^, and 11 were selected from the recent GWAS^6^. They were analyzed in the largest collection of patients with RA studied to date and with a focus in reproducibility of the results^22^. A SNP in *MTRR*, rs1801394, showed robust association. None of the other SNPs reached our demanding standards.

## Materials and Methods

### Patients

The study population consisted of 956 patients with RA according to the 1987 American College of Rheumatology criteria^23^. They were of European Caucasian origin, and more specifically of Spanish (269), Slovenian (103), Portuguese (230) and Polish (354) origin. The patients were recruited with written informed consent. The study was approved by the Autonomous Research Ethics Committee of Galicia (Ref. 2014/387). All protocols and methods were conducted according with the relevant guidelines (Declaration of Helsinki and the Belmont Report) and regulations (Spanish Law 14/2007 of Biomedical Research). All patients have received MTX monotherapy for 6 months. Evaluations included Disease Activity Score 28 (DAS28) at the start of treatment and at 6 months. DAS28 is a composite index of RA activity including the number of tender joints and swollen joints (28 joints maximum), erythrocyte sedimentation rate, and global patient health status assessment^24^. DAS28, at the start of the treatment and at 6 months, sex and age were used for all analyses. This information was incomplete in 41 patients that were excluded. Main clinical characteristics of the remaining 915 patients are summarized in Table 1.

**Table 1:** Characteristics of the 915 patients with RA and their association with response to MTX.

Characteristic	Value	*P*_ΔDAS28_^a^	*P*_NR_^b^
Women, N (%)	738 (80.6)	0.002	ns
Age at treatment, mean ± SD	52.7 ± 13.7	<10^−5^	0.0003
RF positive, N (%)	555 (60.6)	ns	ns
Anti-CCP positive^c^, N (%)	501 (54.7)	0.001	0.03
Seropositivity (RF or antiCCP)	682 (74.5)	0.002	ns
Erosive arthritis, N (%)	479 (52.3)	<10^−6^	<10^−5^
Smoking habit^c^, N (%)	195 (21.3)	ns	0.003
Previous DMARD, N (%)	185 (20.2)	<10^−6^	<10^−5^
MTX maximum dose^c^, mean ± SD	18.7 ± 4.6	ns	ns
Concomitant treatment			
Corticosteroids, N (%)	660 (72.1)	0.01	0.01
NSAIDs^c^, N (%)	490 (53.5)	0.0004	<10^−5^
Baseline DAS28, mean ± SD	5.3 ± 1.2	<10^−6^	<10^−4^
DAS28 at 6 months, mean ± SD	4.0 ± 1.5		
EULAR response at 6 months, N (%)			
Good responder	250 (27.3)		
Moderate responder	354 (38.7)		
Non-responder	311 (34.0)		

^a^Response to MTX assessed as ΔDAS28 and analyzed with linear regression.

^b^Response to MTX assessed as EULAR non-response (NR) and analyzed with logistic regression.

^c^Data from <85% of the patients were available.

### SNP selection and testing

We selected 25 SNP for study (Supplementary Table S1 for full details and references): 14 SNPs from candidate gene studies, and 11 SNPS from a GWAS. Genotypes were determined by PCR amplification followed by single-base extension with the SNaPshot Multiplex Kit (Applied Biosystems, Foster City, California). The corresponding primers, probes and detailed protocols are available from the authors. Quality control of the results included duplicate assays of 10% of the samples, genotype call rate ≥95%, concordance with the Hardy–Weinberg equilibrium (p > 0.01) and with the SNP frequencies in the HapMap European collection (Utah residents with Northern and Western European ancestry and Iberian populations in Spain taken from http://browser.1000genomes.org/index.html). One SNP (rs1051266) was excluded due to low call rate.

### Statistical analyses

Response to MTX was evaluated as change in DAS28 at 6 months of follow-up (ΔDAS28 = DAS28baseline − DAS28follow-up) as the primary outcome. In addition, non-responder classification according to the European League Against Rheumatism (EULAR) criteria was also assessed^25^. The EULAR criteria divide patients into three classes based on change in DAS28 from baseline (ΔDAS28) and DAS28 at the time of evaluation: good responders are those with ΔDAS28 ≥ 1.2 and DAS28 ≤ 3.2; non-responders (NR) are all patients with ΔDAS28 ≤ 0.6 and those with ΔDAS28 > 0.6 but ≤1.2 and with DAS28 > 5.1; all the remaining patients are moderate responders. Here, good and moderate responders were grouped as responders (n = 604) to confront with the NR (n = 311). Linear and logistic regression models for ΔDAS28 and EULAR NR were fitted, respectively. Genotypes were considered in accordance with an additive genetic model of minor allele counts (0, 1 or 2). Therefore, positive regression coefficients indicate a better response associated with minor allele additive effects. Effect of each SNP was adjusted for sex, age, DAS28 at baseline, treatment with a previous DMARD and concomitant treatment with corticosteroids. In a second analysis, additional putative confounders were included: seropositivity and smoking status. Data from the four populations were combined by individual participant data metaanalysis applying two-stage methods. They involved estimation of the association parameters for each population as the first stage and the combination of the results of the four populations in the second stage. A main effects linear regression model for each population was applied to ΔDAS28. The EULAR outcome, in turn, was assessed with separate logistic regression for each population. The combination was done as fixed effects, weighting the effect corresponding to each population with the inverse variance method, and as random effects, according to DerSimonian and Laird. The fixed effects results were preferred except when heterogeneity was moderate (I^2^ > 50), in which case the random effects metaanalysis was selected. Heterogeneity of effect sizes between populations was assessed with Cochran Q test and the inconsistency parameter I^2^. Results were interpreted considering the number of tests following the Bonferroni approach, concordance of results with the two treatment outcomes, independence from confounding variables, and previous evidence of association with response to MTX. Statistica 7.0 (Statsoft, Tulsa, OK, USA) was used for analyses. Post-hoc power analysis was used to assess the study capacity to replicate previous findings. The effect size to replicate was taken from previous studies, considering the EULAR response because this is the most frequently reported outcome in previous studies. The power analysis was conducted using the GPower NT software for the most extreme reported OR^26^.

### Data availability

The datasets generated during the current study are available from the corresponding author on reasonable request

## Results

The 915 RA patients were predominantly female (81%) and DMARD-naïve (80%) with a mean age of 53 years at the start of MTX monotherapy (Table 1). According to baseline DAS28, many patients presented a high disease activity (DAS28 > 5.1) and most of them received concomitant treatment with corticosteroids or NSAIDs (72.1% and 53.5%, respectively). After 6 months on MTX, only 27.3% of the patients showed a good response according to EULAR criteria, 38.7% showed a moderate response and 34.0% were NR. Several of the clinical and demographic variables were significantly associated with ΔDAS28 or with NR (Table 1). Specifically, men, seronegative patients, patients lacking erosions, not DMARD-experienced or not receiving concomitant corticosteroids showed larger decrease in DAS28 than the corresponding alternative patient subgroups. Associations with NR were similar (Table 1).

**Figure 1:**
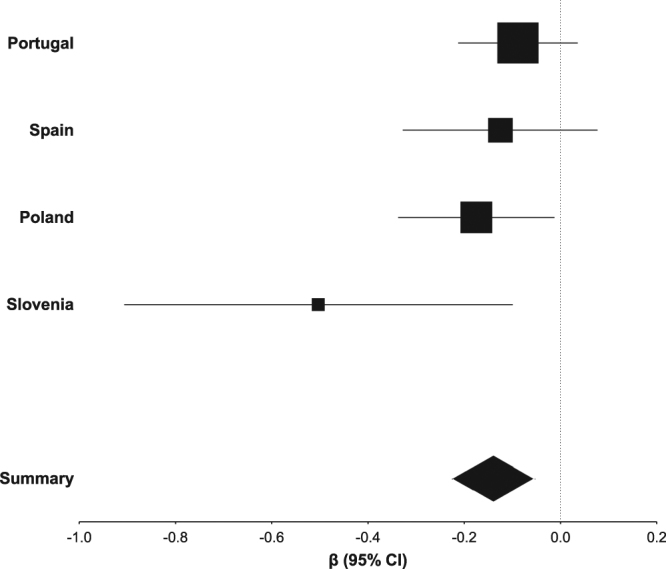
Forest plot of the fixed effects metaanalysis for association of rs1801394 with ΔDAS28 in patients with RA treated with MTX. Square areas are proportional to the weight of the population according to the inverse variance method. Width of the diamond represents the 95% CI of the summary regression coefficient.

After genotyping and quality control, one of the 25 SNPs was discarded. Fixed effect metaanalysis of the nested models on the remaining 24 SNPs showed nominal association with ΔDAS28 (Table 2) of two SNPs from candidate genes (rs2295553 and rs1801394). Only rs1801394 in *MTRR* showed association withstanding Bonferroni correction (*P* = 0.0016). Its minor allele (A) was associated with a lower decrease in DAS28 after MTX treatment than the major allele, specifically, 0.14 less DAS28 units per each A allele according with the additive model. The magnitude of change showed a low level of heterogeneity between the four populations (I^2^ = 24%) that was not significant according with the Q test (P = 0.3) and did not reflect any widely divergent result (Fig. 1).

**Table 2:** Metaanalysis of the association of the 24 SNPs with response to MTX assessed as ΔDAS28 at 6 months in the four populations of RA patients.

Locus	SNP	MA	Fixed effects		Heterogeneity		Random effects	
			β (SE)	*P*	I^2^	*P*	β (SE)	*P*
Candidate gene								
*ABCB1*	rs1045642	A	0.02 (0.04)	0.6	28	0.2	0.02 (0.05)	0.7
*ABCC1*	rs35592	C	0.03 (0.05)	0.6	71	0.02	−0.04 (0.10)	0.7
*AMPD1*	rs17602729	A	−0.11 (0.07)	0.10	61	0.05	−0.17 (0.12)	0.15
*ATIC*	rs4673990	G	0.08 (0.04)	0.07	6	0.4	0.08 (0.05)	0.09
	rs12995526	A	0.04 (0.04)	0.4	36	0.2	0.02 (0.06)	0.7
	rs16853834	T	0.04 (0.06)	0.6	11	0.3	0.04 (0.07)	0.6
	rs2372536	G	0.07 (0.05)	0.1	17	0.3	0.07 (0.06)	0.2
*ITPA*	rs2295553	C	0.09 (0.04)	0.040	0	0.6	0.09 (0.04)	0.040
	rs1127354	A	0.07 (0.09)	0.4	19	0.3	0.06 (0.10)	0.5
*MTRR*	rs1801394	A	−0.14 (0.04)	0.0016	24	0.3	−0.15 (0.05)	0.0056
*MTHFD1*	rs2236225	A	−0.06 (0.05)	0.2	0	0.8	−0.06 (0.05)	0.2
*SLC46A1*	rs2239907	T	−0.01 (0.04)	0.7	0	0.6	−0.01 (0.04)	0.7
*TYMS*	rs699517	T	−0.03 (0.05)	0.5	57	0.07	0.00 (0.08)	1.0
GWAS hit								
*ARL14/PPM1L*	rs7624766	G	−0.03 (0.05)	0.5	0	0.5	−0.03 (0.05)	0.5
*BMP2*	rs2650972	T	−0.04 (0.04)	0.4	0	0.8	−0.04 (0.04)	0.4
*DHFR*	rs5836788	del	−0.01 (0.05)	0.8	0	0.5	−0.01 (0.05)	0.8
*10p15.1*	rs1901633	G	0.05 (0.05)	0.3	0	0.7	0.05 (0.05)	0.3
*14q13.1*	rs4982133	A	−0.06 (0.05)	0.3	0	1.0	−0.06 (0.05)	0.3
*15q26.2*	rs1703794	C	−0.01 (0.05)	0.8	0	0.4	−0.01 (0.05)	0.8
*20q13*	rs6064463	C	−0.06 (0.05)	0.2	11	0.3	−0.06 (0.05)	0.3
*MTRR*	rs162040	C	−0.07 (0.06)	0.3	60	0.06	−0.07 (0.11)	0.5
*PTPRM*	rs6506569	C	0.02 (0.05)	0.6	0	0.8	0.02 (0.05)	0.6
*TYMS*	rs2244500	G	0.02 (0.05)	0.6	64	0.04	0.07 (0.09)	0.4
	rs2847153	A	0.02 (0.06)	0.7	0	0.5	0.02 (0.06)	0.7

Summary regression coefficients (β) and their standard errors (SE) obtained with fixed effects and random effects metaanalysis are presented together with the inconsistency (I^2^) and *P* value of the Cochran Q test for heterogeneity. Multivariate linear regression in each population included sex, age, baseline DAS28, previous use of DMARD and concomitant treatment with corticosteroids.

The previous results were obtained with adjustment by five clinical variables (sex, age, baseline DAS28, previous DMARD treatment and concomitant corticosteroids), which were available from most of the 915 patients. Multivariate analysis adjusting for two other variables associated with response to MTX (smoking and seropositivity) was only possible in 643 patients (Supplementary Table S2). Therefore, the results were compared at the level of effect sizes, which are independent of the number of samples, not at the level of *P* values, which are very sensitive to the number of patients. The effect size of rs1801394 was almost unaltered (from β = −0.14 to −0.13; *P* = 0.009) after the complete adjusted analysis. No other SNP than rs1801394 showed association (*P* < 0.05) in this set of analyses.

Consideration of the secondary outcome of response to MTX, NR according to EULAR criteria, showed a consistent association of rs1801394 (Table 3). The A allele showed OR = 1.39 for NR (95% CI 1.11–1.75) without detectable heterogeneity between the populations (I^2^ = 0). There were three other SNPs showing nominal association (rs4673990, rs2372536 and rs2650972) with little heterogeneity between the populations. As for the primary outcome, inclusion of more variables of adjustment did not modify the effect size of rs1801394 (from OR = 1.39 to 1.37) reinforcing the evidence of its independent association with MTX response (Supplementary Table S2). Two of the other SNPs showing association with NR were reinforced in the fully adjusted analysis (rs4673990 and rs2372536), but at the expense of increased heterogeneity between the populations.

**Table 3:** Metaanalysis of the association of the 24 SNPs with response to MTX according to the EULAR criteria at 6 months in the four populations of RA patients.

Locus	SNP	MA	Fixed effects		Heterogeneity		Random effects	
			OR (95% CI)	*P*	I^2^	*P*	OR (95% CI)	*P*
Candidate gene								
*ABCB1*	rs1045642	A	0.95 (0.8–1.2)	0.6	22	0.3	0.97 (0.7–1.2)	0.8
*ABCC1*	rs35592	C	1.19 (0.9–1.5)	0.2	61	0.05	1.24 (0.8–1.9)	0.3
*AMPD1*	rs17602729	A	1.08 (0.8–1.5)	0.6	42	0.2	1.14 (0.7–1.8)	0.6
*ATIC*	rs4673990	G	0.73 (0.6–0.9)	0.009	0	0.5	0.73 (0.6–0.9)	0.009
	rs12995526	A	0.85 (0.7–1.1)	0.1	52	0.10	0.86 (0.6–1.2)	0.4
	rs16853834	T	0.91 (0.7–1.3)	0.6	0	0.5	0.91 (0.7–1.3)	0.6
	rs2372536	G	0.72 (0.6–0.9)	0.009	8	0.4	0.72 (0.6–0.9)	0.014
*ITPA*	rs2295553	C	0.81 (0.6–1.02)	0.1	0	1.0	0.81 (0.6–1.02)	0.1
	rs1127354	A	0.74 (0.5–1.1)	0.2	0	0.6	0.74 (0.5–1.1)	0.2
*MTRR*	rs1801394	A	1.39 (1.1–1.8)	0.004	0	0.6	1.39 (1.1–1.8)	0.004
*MTHFD1*	rs2236225	A	1.03 (0.8–1.3)	0.8	0	0.5	1.03 (0.8–1.3)	0.8
*SLC46A1*	rs2239907	T	0.96 (0.8–1.2)	0.7	0	0.8	0.96 (0.8–1.2)	0.7
*TYMS*	rs699517	T	1.03 (0.8–1.3)	0.8	0	0.6	1.03 (0.8–1.3)	0.8
GWAS hit								
*ARL14/PPM1L*	rs7624766	G	1.13 (0.9–1.4)	0.3	47	0.13	1.15 (0.8–1.6)	0.4
*BMP2*	rs2650972	T	1.28 (1.02–1.6)	0.035	0	0.8	1.28 (1.02–1.6)	0.035
*DHFR*	rs5836788	del	1.00 (0.8–1.3)	1.0	17	0.3	1.00 (0.8–1.3)	1.0
*10p15.1*	rs1901633	G	0.88 (0.7–1.1)	0.3	0	0.9	0.88 (0.7–1.1)	0.3
*14q13.1*	rs4982133	A	1.19 (0.9–1.6)	0.2	0	0.7	1.19 (0.9–1.6)	0.2
*15q26.2*	rs1703794	C	1.12 (0.9–1.5)	0.4	5	0.4	1.12 (0.8–1.5)	0.4
*20q13*	rs6064463	C	1.17 (0.9–1.5)	0.2	0	0.5	1.17 (0.9–1.5)	0.2
*MTRR*	rs162040	C	1.35 (0.98–1.9)	0.1	0	1.0	1.35 (0.98–1.9)	0.1
*PTPRM*	rs6506569	C	0.89 (0.7–1.1)	0.3	0	0.5	0.89 (0.7–1.1)	0.3
*TYMS*	rs2244500	G	0.89 (0.7–1.1)	0.4	0	0.9	0.89 (0.7–1.1)	0.4
	rs2847153	A	1.02 (0.8–1.4)	0.9	0	0.7	1.02 (0.8–1.4)	0.9

Summary odds ratios (OR) and their confidence intervals (95% CI) obtained with fixed effects and random effects metaanalysis are presented together with the inconsistency (I^2^) and p value of the Cochran Q test for heterogeneity. Multivariate logistic regression in each population included sex, age, baseline DAS28, previous use of DMARD and concomitant treatment with corticosteroids.

## Discussion

Here, we have identified a SNP, rs1801394, in *MTRR* associated with response to MTX with all the characteristics we had specified in advance to increase reproducibility^22^. This focus in reproducibility differs from many previous studies on the same field^3–5^. The remaining 23 SNPs from candidate gene studies or from GWAS did not show association at these high standards. These results have the added value of arising from the largest collection of patients analyzed to date in the genetics of response to MTX.

The association of rs1801394 (also called A66G) with response to MTX fulfilled all the requirements of our analysis: its *P* value was low enough to allow for correction by the number of tests, it was consistently observed with the two treatment outcomes (although the NR *P* value was over the corrected threshold), ΔDAS28 and EULAR criteria, and it was independent of the other variables with influence on response to MTX, including differences in disease activity, treatment and sample collection. These requisites were considered necessary to increase reproducibility, which is critical for validation of any biomarker^3–5,22^.

The uncritical use of *P* < 0.05 for claiming association has been shown to lead to many false positive results. Correction by the number of tests, although not without limitations, is widely considered to be very beneficial in genetics of complex diseases^22,27^. Other characteristic that distinguishes our analysis is the adjustment for multiple potential confounding variables. Variables like the baseline level of disease activity or the previous use of DMARDs could have a different distribution in patients with different genotypes and introduce bias in the results as they are strongly associated with MTX response. Another distinctive characteristic of our study is analysis according to the additive genetic model with values 0, 1 and 2 for the number of minor alleles. In selecting the additive model we have followed the approach of the GWAS that have chosen the additive model based in its biological plausibility and statistical reproducibility^27^.

Less consensus exists in the outcome that should be used in the search of biomarkers of response to treatment in RA. ΔDAS28 has all the advantages of quantitative traits in comparison with dichotomous traits for statistics: discrimination, robustness, increased power and reproducibility^28,29^. These considerations motivated our use of ΔDAS28 as the primary outcome for analysis, which is concordant with the large studies and joint initiatives analyzing response to biologic DMARDs^30–32^. The only advantage of dichotomous outcomes is its simplicity. However, this simplicity is misleading because definitions of NR vary widely between studies. In effect, some authors have used the EULAR criteria of response, others have used DAS28-based remission, or low-disease activity, or the ACR 20 and ACR 50 response criteria, and even study-specific definitions of response^4^. As a consequence, it is suspected that heterogeneity in these definitions could contribute to lack of reproducibility. In any case, we decided to include the EULAR criteria as a secondary outcome for completeness.

All the discussed characteristics were intended to increase the reproducibility of our results. However, the association of response to MTX with rs1801394 in *MTRR* is not yet certain. Additional support could be obtained from its credibility as a good candidate^22^. This SNPs causes the substitution of isoleucine for methionine at amino acid 22 of MTRR, 5-methyltetrahydrofolate-homocysteine methyltransferase reductase. This enzyme collaborates with MTR (5-methyltetrahydrofolate-homocysteine methyltransferase) in the transfer of a methyl group from 5-methy tetra-hydro-folic acid to homocysteine, resulting in de novo production of L-methionine and tetra-hydro-folic acid (in a reaction involving vitamin B12 as cofactor). This step in the folic acid cycle precedes the production of the active form of L-methionine, S-adenosyl-methionine, which is the methyl donor in hundreds of biologic transmethylation reactions and the donor of propylamine in polyamine synthesis. Specifically, the A allele, herein associated with poor response to MTX, has been related to a moderate decrease in total plasma levels of homocysteine, which is interpreted as increased capacity to regenerate methionine^33^. More pertinent for our study, patients with RA under MTX treatment displayed folate levels in red blood cells (RBC) that were higher in carriers of the rs1801394 A allele compared to the G/G genotype^16^. This relationship between MTRR and intracellular folate could be linked with the association we have observed because RA patients displaying a decrease in RBC folate (in the form of folate polyglutamates) concentration during MTX treatment showed a marked decrease of DAS28 at 6 months^18^. This indirect relationship seems to indicate that the A allele could decrease response to MTX by leading to increased intracellular folate, which is a factor already suspected due to the interference of folic or folinic acid supplementation with MTX treatment^34–36^. However, the more direct evidence linking rs1801394 with response to MTX comes from previous genetic studies. There have been three studies analyzing rs1801394 as a biomarker of response to MTX in patients with RA^18,19,37^, and two in patients with juvenile idiopathic arthritis (JIA)^38,39^. Two of these studies, one in RA^19^, and one in JIA^38^ have found the same association reported here. The negative studies were either smaller (n = 48 in^18^, and 119 in^39^) or from a different ethnic background (n = 322 Indians in^37^). As the RA study that has found association overlaps with our study^19^, we analyzed the association between rs1801394 and treatment response after excluding the overlapping collection of patients, finding similar effect sizes (n = 664 patients; ΔDAS28 β = −0.19, SE = 0.06, *P* = 0.002; NR OR = 1.33, 95% CI = 1.03–1.7, *P* = 0.030) to those obtained in the total population. Therefore, we can conclude that previous evidence supports a functional relationship of rs1801394 with alterations in the folate pathway and provide suggestive support of its association with response to treatment, although further validation is still needed.

In this study, we analyzed other 23 SNPs from previous candidate gene studies and from a GWAS (Supplementary Table S1). However, none showed an association below the corrected threshold or was associated with the two outcomes at the nominal level of P < 0.05. In addition, three of the four that showed nominal association in any analysis, rs2295553, rs4673990 and rs2372536, were in the opposite direction to previous reports. The only concordant SNP, rs2650972, was just below the 0.05 level for the EULAR outcome (P = 0.035) and far over this threshold for ΔDAS28. This lack of reproducibility is common for candidate gene studies, as signaled by previous systematic revisions and meta-analysis^3–5^. The causes are many, but it is worth to highlight that the candidate gene approach responds to a concept of complex traits that has been demonstrated overly optimistic. In fact, progress in genetics has shown that many complex traits have very polygenic causality with many involved loci, each of them with very small contribution. Therefore, the candidate gene approach has been almost abandoned in favor of GWAS, which are done in much larger sample collections and with strict quality control and requirements to claim association^27^. Unfortunately, the new requirements are hard to fulfill in areas like the response to treatment in RA. This is exemplified by the study of response to TNF inhibitors, where only after multiple consortia it was possible to put together about 3000 patients^30^. The GWAS on response to MTX in comparison only included 457 patients and none of the highlighted loci reached the threshold of significance set for this type of studies^6^. Therefore, it is not surprising that we did not replicate any of the SNPs selected from that GWAS in our study.

Our study shows some limitations that include lack of complete information from all patients, possible confounding by indication common to retrospective studies without randomization, the requirement of 6 months on MTX and insufficient power to detect associations with small effect sizes, which are common in complex traits. However, statistical power of our study was sufficient to replicate previous published effect sizes. Only a SNP did not show power >0.8 to replicate previously findings with *P* < 0.05. This SNP was rs162040, which showed power = 0.71 for detecting association with *P* < 0.05. As the OR of rs162040 in our study was 1.35 without heterogeneity and the OR in the previous study was 1.45 and with similar increase in NR associated with the minor allele^6^, it is possible that lack of power has been an issue in our results for this SNP. This possibility is interesting because rs162040 is also in the *MTRR* locus. The next weakest power was obtained for rs2847153 that showed power = 0.82 for association with *P* < 0.05. However, in this case the effect size in our samples was so low (OR = 1.02) that lack of power did not seem relevant. All the other SNPs showed power ≥0.79 for replication of published effects with *P* < 0.01. Therefore, the association of rs1801394 needs to be replicated in other context not only to confirm its accuracy, but also to know if it extends to other patient groups, including other ethnic backgrounds, and study designs.

In conclusion, our results show only a SNP, rs1801394, in *MTRR* with a robust association with response to MTX monotherapy among the 24 analyzed. This association surpassed all the requirements we have imposed aiming to increase reproducibility and was supported by the study sample size, the largest to date on MTX treatment, and suggestive evidence about the SNP function and previous association data. However, it still needs confirmation. It is to be expected this finding could stimulate further collaborations and efforts to progress in the search of biomarkers predicting the important outcome of MTX treatment.

## Electronic supplementary material

Supplementary Tables
