# Supplementary material for: Replication study of polymorphisms associated with response to methotrexate in patients with rheumatoid arthritis
Source: Sci Rep. 2018 May 9;8:7342. doi: 10.1038/s41598-018-25634-y (PMC5943457; doi:10.1038/s41598-018-25634-y)
Supplement: Supplementary file 1 — Supplementary Tables [file 41598_2018_25634_MOESM1_ESM.doc]

**Supplementary information:**

**Replication study of polymorphisms associated with response to methotrexate in patients with rheumatoid arthritis**

Rosario López-Rodríguez ^1^, Aida Ferreiro-Iglesias ^1^, Aurea Lima ^2^, Miguel Bernardes ^3^, Andrzej Pawlik ^4^, Agnieszka Paradowska-Gorycka ^5^, Jerzy Świerkot ^6^, Ryszard Slezak ^7^, [Vita Dolžan](http://www.frontiersin.org/people/u/21502) ^8^, Isidoro Gonzalez-Alvaro ^9^, Javier Narvaez ^10^, Rafael Caliz ^11^, Eva Pérez-Pampín ^1^, Antonio Mera-Varela ^1^, Laura Vidal-Bralo ^1^, José Gorgonio Acuña Ochoa ^1^, Carmen Conde ^1^, Juan J. Gómez-Reino ^1^, Antonio González ^1*^.

^1^ Experimental and Observational Rheumatology and Rheumatology Unit. Instituto de Investigación Sanitaria - Hospital Clínico Universitario de Santiago, Santiago de Compostela, Spain

^2^ CESPU, Institute of Research & Advanced Training in Health Sciences & Technologies, Department of Pharmaceutical Sciences, Gandra PRD, Portugal

^3^ Faculty of Medicine of University of Porto, Porto, Portugal. Rheumatology Department of São João Hospital Center, Porto, Portugal

^4^ Department of Physiology Pomeranian Medical University Szczecin, Poland

^5^ Department of Biochemistry and Molecular Biology, National Institute of Geriatrics, Rheumatology and Rehabilitation, Warsaw, Poland

^6^ Department of Rheumatology, Wroclaw Medical University, Wroclaw, Poland

^7^ Department of Genetics, Medical University of Wroclaw, Wroclaw, Poland

**^8^** Pharmacogenetics Laboratory, Institute of Biochemistry, Faculty of Medicine, University of Ljubljana Ljubljana, Slovenia

^9^ Rheumatology Department. Instituto de Investigacion del Hospital de La Princesa (IIS-IP), Madrid, Spain

^10^ Department of Rheumatology, Hospital Universitario de Bellvitge-IDIBELL, Barcelona, Spain

^11^ Rheumatology Unit, Hospital Universitario Virgen de las Nieves, Granada, Spain

*** Correspondence and reprint request to AG:** agmartinezp@ser.es

**Supplementary Table S1.** Information on the SNPs included in the study, including the frequency or their minor alleles (%) and the *P* value of concordance with the Hardy-Weinberg equilibrium (HWE) in our 915 patients with RA.

| Locus | HGVS Name | SNP | Ref. | Minor  allele | % | HWE  *P* |
| --- | --- | --- | --- | --- | --- | --- |
| Candidate gene |  |  |  |  |  |  |
| *ABCB1* | NM_000927.4:c.3435T>C | rs1045642 | [1–3] | A | 47 | 0.14 |
| *ABCC1* | NM_004996.3:c.1219-176T>C | rs35592 | [3] | C | 25 | 0.15 |
| *AMPD1* | NM_000036.2:c.133C>T | rs17602729 | [4,5] | A | 13 | 0.54 |
| *ATIC* | NM_004044.6:c.1227+2423A>G | rs4673990 | [6] | G | 46 | 0.59 |
|  | NM_004044.6:c.815-102T>C | rs12995526 | [6,7] | C | 50 | 0.01 |
|  | NM_004044.6:c.1320+642C>T | rs16853834 | [7] | T | 16 | 1.00 |
|  | NM_004044.6:c.347C>G | rs2372536 | [4,5] | G | 33 | 0.29 |
| *ITPA* | NM_023935.2:c.92-72G>A | rs2295553 | [6] | C | 43 | 0.19 |
|  | NM_001267623.1:c.67-789C>A | rs1127354 | [4,5] | A | 7 | 0.32 |
| *MTRR* | NM_002454.2:c.66A>G | rs1801394 | [8,9] | A | 46 | 0.46 |
| *MTHFD1* | NM_005956.3:c.1958G>A | rs2236225 | [4,5] | A | 43 | 0.95 |
| *SLC46A1* | NM_001242366.2:c.*928A>G | rs2239907 | [10] | T | 44 | 0.73 |
| *SLC19A1* | NM_001205206.1:c.80A>G | rs1051266^a^ | [8,10–12] | T | 44 | 0.88 |
| *TYMS* | NM_001071.2:c.*19C>T | rs699517 | [11] | T | 30 | 0.75 |
| GWAs hits |  |  |  |  |  |  |
| *ARL14/PPM1L* | [NC_000003](http://www.ensembl.org/Homo_sapiens/Location/View?contigviewbottom=variation_feature_variation%3Dnormal;db=core;source=dbSNP;v=rs7624766;vdb=variation;vf=4586690).12:g.160712081A>G | rs7624766 | [13] | G | 40 | 0.47 |
| *BMP2* | [NC_000020](http://www.ensembl.org/Homo_sapiens/Location/View?contigviewbottom=variation_feature_variation%3Dnormal;db=core;source=dbSNP;v=rs2650972;vdb=variation;vf=2005105).11:g.6802627T>C | rs2650972 | [13] | T | 46 | 0.01 |
| *DHFR* | [NC_000002](http://www.ensembl.org/Homo_sapiens/Location/View?contigviewbottom=variation_feature_variation%3Dnormal;db=core;source=dbSNP;v=rs5836788;vdb=variation;vf=3302539).12:g.181437035delC | rs5836788 | [13] | del | 36 | 0.88 |
| *10p15.1* | [NC_000010](http://www.ensembl.org/Homo_sapiens/Location/View?contigviewbottom=variation_feature_variation%3Dnormal;db=core;source=dbSNP;v=rs1901633;vdb=variation;vf=1335251).11:g.4768369A>G | rs1901633 | [13] | G | 31 | 0.15 |
| *14q13.1* | [NC_000014](http://www.ensembl.org/Homo_sapiens/Location/View?contigviewbottom=variation_feature_variation  0normal;db=core;source=dbSNP;v=rs4982133;vdb=variation;vf=3214263).9:g.34057613A>C | rs4982133 | [13] | A | 27 | 0.48 |
| *15q26.2* | [NC_000015](http://www.ensembl.org/Homo_sapiens/Location/View?contigviewbottom=variation_feature_variation  0normal;db=core;source=dbSNP;v=rs1703794;vdb=variation;vf=1195375).10:g.99069944T>C | rs1703794 | [13] | C | 24 | 0.51 |
| *20q13* | [NC_000020](http://www.ensembl.org/Homo_sapiens/Location/View?contigviewbottom=variation_feature_variation%3Dnormal;db=core;source=dbSNP;v=rs6064463;vdb=variation;vf=3486602).11:g.56905221T>C | rs6064463 | [13] | C | 41 | 0.47 |
| *MTRR* | NM_002454.2:c.1146+662C>A | rs162040 | [13] | C | 14 | 0.18 |
| *PTPRM* | NM_001105244.1:c.2755-20509T>C | rs6506569 | [13] | C | 49 | 0.45 |
| *TYMS* | NM_001071.2:c.280-1141A>G | rs2244500 | [13] | G | 44 | 0.67 |
|  | NM_001071.2:c.280-499G>A | rs2847153 | [13] | A | 20 | 0.83 |

^a^Excluded from the statistical analysis after quality control assessment (rs1051266, call rate < 95%)

**References Supplementary Table S1:**

1. Pawlik A, Wrzesniewska J, Fiedorowicz-Fabrycy I, Gawronska-Szklarz B. The MDR1 3435 polymorphism in patients with rheumatoid arthritis. Int J Clin Pharmacol Ther. 2004;42: 496–503.

2. Drozdzik M, Rudas T, Pawlik A, Kurzawski M, Czerny B, Gornik W, et al. The effect of 3435C>T MDR1 gene polymorphism on rheumatoid arthritis treatment with disease-modifying antirheumatic drugs. Eur J Clin Pharmacol. 2006;62: 933–7. doi:10.1007/s00228-006-0192-1

3. Kooloos WM, Wessels JA, van der Straaten T, Allaart CF, Huizinga TW, Guchelaar H-J. Functional polymorphisms and methotrexate treatment outcome in recent-onset rheumatoid arthritis. Pharmacogenomics. 2010;11: 163–75. doi:10.2217/pgs.09.139

4. Fransen J, Kooloos WM, Wessels JAM, Huizinga TWJ, Guchelaar H-J, van Riel PLCM, et al. Clinical pharmacogenetic model to predict response of MTX monotherapy in patients with established rheumatoid arthritis after DMARD failure. Pharmacogenomics. 2012;13: 1087–94. doi:10.2217/pgs.12.83

5. Wessels JAM, Van Der Kooij SM, Le Cessie S, Kievit W, Barerra P, Allaart CF, et al. A clinical pharmacogenetic model to predict the efficacy of methotrexate monotherapy in recent-onset rheumatoid arthritis. Arthritis Rheum. 2007;56: 1765–1775. doi:10.1002/art.22640

6. Hinks A, Moncrieffe H, Martin P, Ursu S, Lal S, Kassoumeri L, et al. Association of the 5-aminoimidazole-4-carboxamide ribonucleotide transformylase gene with response to methotrexate in juvenile idiopathic arthritis. Ann Rheum Dis. 2011;70: 1395–400. doi:10.1136/ard.2010.146191

7. Owen S, Hider S, Martin P, Bruce I, Barton A, Thomson W. Genetic polymorphisms in key methotrexate pathway genes are associated with response to treatment in rheumatoid arthritis patients. Pharmacogenomics J. 2012;13: 227–234. doi:10.1038/tpj.2012.7

8. Kato T, Hamada A, Mori S, Saito H. Genetic polymorphisms in metabolic and cellular transport pathway of methotrexate impact clinical outcome of methotrexate monotherapy in Japanese patients with rheumatoid arthritis. Drug Metab Pharmacokinet. 2012;27: 192–9.

9. Stamp LK, Chapman PT, O’Donnell JL, Zhang M, James J, Frampton C, et al. Polymorphisms within the folate pathway predict folate concentrations but are not associated with disease activity in rheumatoid arthritis patients on methotrexate. Pharmacogenet Genomics. 2010;20: 367–76. doi:10.1097/FPC.0b013e3283398a71

10. James HM, Gillis D, Hissaria P, Lester S, Somogyi AA, Cleland LG, et al. Common polymorphisms in the folate pathway predict efficacy of combination regimens containing methotrexate and sulfasalazine in early rheumatoid arthritis. J Rheumatol. 2008;35: 562–71.

11. Drozdzik M, Rudas T, Pawlik A, Gornik W, Kurzawski M, Herczynska M. Reduced folate carrier-1 80G 4 A polymorphism affects methotrexate treatment outcome in rheumatoid arthritis. Pharmacogenomics J. 2007;7: 404–407. doi:10.1038/sj.tpj.6500438

12. Dervieux T, Greenstein N, Kremer J. Pharmacogenomic and metabolic biomarkers in the folate pathway and their association with methotrexate effects during dosage escalation in rheumatoid arthritis. Arthritis Rheum. 2006;54: 3095–103. doi:10.1002/art.22129

13. Senapati S, Singh S, Das M, Kumar A, Gupta R, Kumar U, et al. Genome-wide analysis of methotrexate pharmacogenomics in rheumatoid arthritis shows multiple novel risk variants and leads for TYMS regulation. Pharmacogenet Genomics. 2014;24: 211–9. doi:10.1097/FPC.0000000000000036

**Supplementary Table S2.** Association with response to MTX as change in DAS28 (ΔDAS28) adjusted for smoking and seropositivity in addition to concomitant treatment with corticosteroids, previous DMARD use, sex, age and DAS28 at baseline. A total of the 643 patients with RA were available for these analyses, which excluded Slovenia patients due to missing data. Results are presented as in Table 2 of the main text.

|  |  |  | Fixed effects | | Heterogeneity | | Random effects | |
| --- | --- | --- | --- | --- | --- | --- | --- | --- |
| Locus | SNP | MA | β (SE) | *P* | I^2^ | *P* | β (SE) | *P* |
| Candidate gene |  |  |  |  |  |  |  |  |
| *ABCB1* | rs1045642 | A | 0.04 (0.05) | 0.4 | 1 | 0.4 | 0.04 (0.05) | 0.4 |
| *ABCC1* | rs35592 | C | 0.08 (0.05) | 0.14 | 0 | 0.7 | 0.08 (0.05) | 0.14 |
| *AMPD1* | rs17602729 | A | -0.11 (0.07) | 0.14 | 0 | 0.5 | -0.11 (0.07) | 0.14 |
| *ATIC* | rs4673990 | G | 0.07 (0.05) | 0.15 | 57 | 0.10 | 0.07 (0.08) | 0.39 |
|  | rs12995526 | A | 0.04 (0.05) | 0.4 | 46 | 0.2 | 0.02 (0.07) | 0.7 |
|  | rs16853834 | T | 0.02 (0.07) | 0.8 | 3 | 0.4 | 0.02 (0.07) | 0.8 |
|  | rs2372536 | G | 0.06 (0.05) | 0.3 | 29 | 0.2 | 0.05 (0.07) | 0.4 |
| *ITPA* | rs2295553 | C | 0.07 (0.05) | 0.15 | 0 | 0.4 | 0.07 (0.05) | 0.15 |
|  | rs1127354 | A | 0.09 (0.1) | 0.4 | 0 | 0.5 | 0.09 (0.1) | 0.4 |
| *MTRR* | rs1801394 | A | -0.13 (0.05) | 0.009 | 0 | 0.6 | -0.13 (0.05) | 0.009 |
| *MTHFD1* | rs2236225 | A | -0.06 (0.05) | 0.2 | 0 | 0.9 | -0.06 (0.05) | 0.2 |
| *SLC46A1* | rs2239907 | T | -0.02 (0.05) | 0.7 | 0 | 0.7 | -0.02 (0.05) | 0.7 |
| *TYMS* | rs699517 | T | -0.03 (0.05) | 0.5 | 68 | 0.05 | 0.02 (0.1) | 0.9 |
| GWAS hit |  |  |  |  |  |  |  |  |
| *ARL14/PPM1L* | rs7624766 | G | 0.00 (0.05) | 0.9 | 0 | 0.5 | 0.00 (0.05) | 0.9 |
| *BMP2* | rs2650972 | T | -0.03 (0.05) | 0.5 | 0 | 0.6 | -0.03 (0.05) | 0.5 |
| *DHFR* | rs5836788 | del | 0.03 (0.05) | 0.5 | 0 | 0.9 | 0.03 (0.05) | 0.5 |
| *10p15.1* | rs1901633 | G | 0.06 (0.05) | 0.3 | 0 | 0.7 | 0.06 (0.05) | 0.3 |
| *14q13.1* | rs4982133 | A | -0.05 (0.06) | 0.4 | 0 | 0.8 | -0.05 (0.06) | 0.4 |
| *15q26.2* | rs1703794 | C | -0.02 (0.06) | 0.8 | 45 | 0.2 | -0.01 (0.08) | 0.9 |
| *20q13* | rs6064463 | C | -0.06 (0.05) | 0.3 | 69 | 0.04 | -0.04 (0.1) | 0.7 |
| *MTRR* | rs162040 | C | -0.05 (0.07) | 0.5 | 44 | 0.2 | -0.05 (0.1) | 0.6 |
| *PTPRM* | rs6506569 | C | 0.02 (0.05) | 0.6 | 0 | 0.9 | 0.02 (0.05) | 0.6 |
| *TYMS* | rs2244500 | G | 0.00 (0.05) | 1.0 | 77 | 0.01 | 0.06 (0.12) | 0.6 |
|  | rs2847153 | A | 0.02 (0.06) | 0.7 | 42 | 0.2 | 0.04 (0.09) | 0.7 |

**Supplementary Table S3.** Association with response to MTX as non-responder (NR) adjusted for smoking and seropositivity in addition to concomitant treatment with corticosteroids, previous DMARD use, sex, age and DAS28 at baseline. A total of 643 patients with RA were available for these analyses, which excluded Slovenia patients due to missing data. Results are presented as in Table 3 of the main text.

|  |  |  | Fixed effects | | Heterogeneity | | Random effects | |
| --- | --- | --- | --- | --- | --- | --- | --- | --- |
| Locus | SNP | MA | OR (95% CI) | *P* | I^2^ | *P* | OR (95% CI) | *P* |
| Candidate gene |  |  |  |  |  |  |  |  |
| *ABCB1* | rs1045642 | A | 0.87 (0.7-1.2) | 0.3 | 27 | 0.3 | 0.87 (0.6-1.2) | 0.4 |
| *ABCC1* | rs35592 | C | 0.93 (0.7-1.3) | 0.7 | 0 | 0.6 | 0.93 (0.7-1.3) | 0.7 |
| *AMPD1* | rs17602729 | A | 1.09 (0.7-1.7) | 0.7 | 11 | 0.3 | 1.08 (0.7-1.7) | 0.7 |
| *ATIC* | rs4673990 | G | 0.67 (0.5-0.9) | 0.007 | 46 | 0.2 | 0.69 (0.5-1.03) | 0.07 |
|  | rs12995526 | A | 0.81 (0.6-1.1) | 0.12 | 63 | 0.07 | 0.83 (0.5-1.3) | 0.4 |
|  | rs16853834 | T | 1.02 (0.7-1.5) | 0.9 | 0 | 0.6 | 1.02 (0.7-1.5) | 0.9 |
|  | rs2372536 | G | 0.70 (0.5-0.96) | 0.025 | 47 | 0.2 | 0.72 (0.5-1.1) | 0.13 |
| *ITPA* | rs2295553 | C | 0.83 (0.6-1.1) | 0.2 | 0 | 0.8 | 0.83 (0.6-1.1) | 0.2 |
|  | rs1127354 | A | 0.72 (0.4-1.3) | 0.3 | 37 | 0.2 | 0.75 (0.4-1.5) | 0.4 |
| *MTRR* | rs1801394 | A | 1.37 (1.03-1.8) | 0.029 | 0 | 0.7 | 1.37 (1.03-1.8) | 0.029 |
| *MTHFD1* | rs2236225 | A | 1.11 (0.8-1.5) | 0.5 | 0 | 0.6 | 1.11 (0.8-1.5) | 0.5 |
| *SLC46A1* | rs2239907 | T | 1.04 (0.8-1.4) | 0.8 | 29 | 0.2 | 1.02 (0.7-1.4) | 0.9 |
| *TYMS* | rs699517 | T | 0.98 (0.7-1.4) | 0.9 | 0 | 0.7 | 0.98 (0.7-1.4) | 0.9 |
| GWAS hit |  |  |  |  |  |  |  |  |
| *ARL14/PPM1L* | rs7624766 | G | 1.00 (0.7-1.3) | 1.0 | 40 | 0.2 | 1.00 (0.7-1.5) | 1.0 |
| *BMP2* | rs2650972 | T | 1.26 (0.96-1.7) | 0.10 | 0 | 0.8 | 1.26 (0.96-1.7) | 0.10 |
| *DHFR* | rs5836788 | del | 0.91 (0.7-1.2) | 0.6 | 40 | 0.2 | 0.92 (0.6-1.4) | 0.7 |
| *10p15.1* | rs1901633 | G | 0.87 (0.6-1.2) | 0.4 | 0 | 0.9 | 0.87 (0.6-1.2) | 0.4 |
| *14q13.1* | rs4982133 | A | 1.20 (0.9-1.7) | 0.3 | 0 | 0.8 | 1.20 (0.9-1.7) | 0.3 |
| *15q26.2* | rs1703794 | C | 1.16 (0.8-1.6) | 0.4 | 51 | 0.13 | 1.21 (0.8-1.9) | 0.4 |
| *20q13* | rs6064463 | C | 1.27 (0.9-1.7) | 0.11 | 13 | 0.3 | 1.26 (0.9-1.7) | 0.15 |
| *MTRR* | rs162040 | C | 1.20 (0.8-1.8) | 0.4 | 0 | 1.0 | 1.20 (0.8-1.8) | 0.4 |
| *PTPRM* | rs6506569 | C | 0.85 (0.6-1.1) | 0.3 | 0 | 0.7 | 0.85 (0.6-1.1) | 0.3 |
| *TYMS* | rs2244500 | G | 0.86 (0.6-1.2) | 0.3 | 0 | 0.6 | 0.86 (0.6-1.2) | 0.3 |
|  | rs2847153 | A | 0.90 (0.6-1.3) | 0.6 | 0 | 0.8 | 0.90 (0.6-1.3) | 0.6 |
